# Lightning scars on tropical trees—Evidence and opportunities

**DOI:** 10.1002/ece3.10210

**Published:** 2023-06-15

**Authors:** Bianca Zoletto, Masha T. van der Sande, Peter van der Sleen, Douglas Sheil

**Affiliations:** ^1^ Forest Ecology and Forest Management Group Wageningen University Wageningen The Netherlands

**Keywords:** Central Africa, lightning, scars, tropical forest

## Abstract

Lightning strikes are a significant cause of tree mortality and damage in some regions of the tropics. Formation of lightning scars on tropical trees, however, is considered rare and therefore of little relevance in identifying trees struck by lightning. Here, we suggest, based on observations made in the Bwindi Impenetrable National Park (Uganda), that lightning scars can be frequent and may be a useful diagnostic feature to aid in identifying trees struck by lightning.

## Graphical abstract

Our study presents novel observations on lightning scars in a tropical montane forest, challenging the previous assumption that these scars are almost absent in the tropics. We argue that lightning scars can be a valuable tool to identify trees struck by lightning and suggest their inclusion as part of the criteria for lightning strike identification in tropical forests.

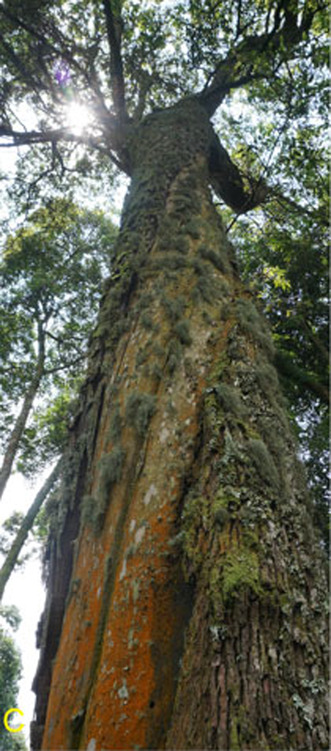

In April 2015, a male silverback gorilla was killed by lightning in the Bwindi Impenetrable National Park, Uganda. The lightning struck the *Faurea saligna* tree the gorilla was under. The dead animal was found the next day by park rangers, who deduced the cause of his death from the long, freshly opened gash along the trunk of the tree. Later, an autopsy on the gorilla confirmed the cause of death as lightning (https://aclenet.org/news‐publications/country‐news/uganda‐injuries/uganda‐2015.html). Eight years later, the tree is alive and still exhibits the scar (Figure 1a). Here, we suggest that the identification of such scars can be used to detect lightning strikes.

**FIGURE 1:**
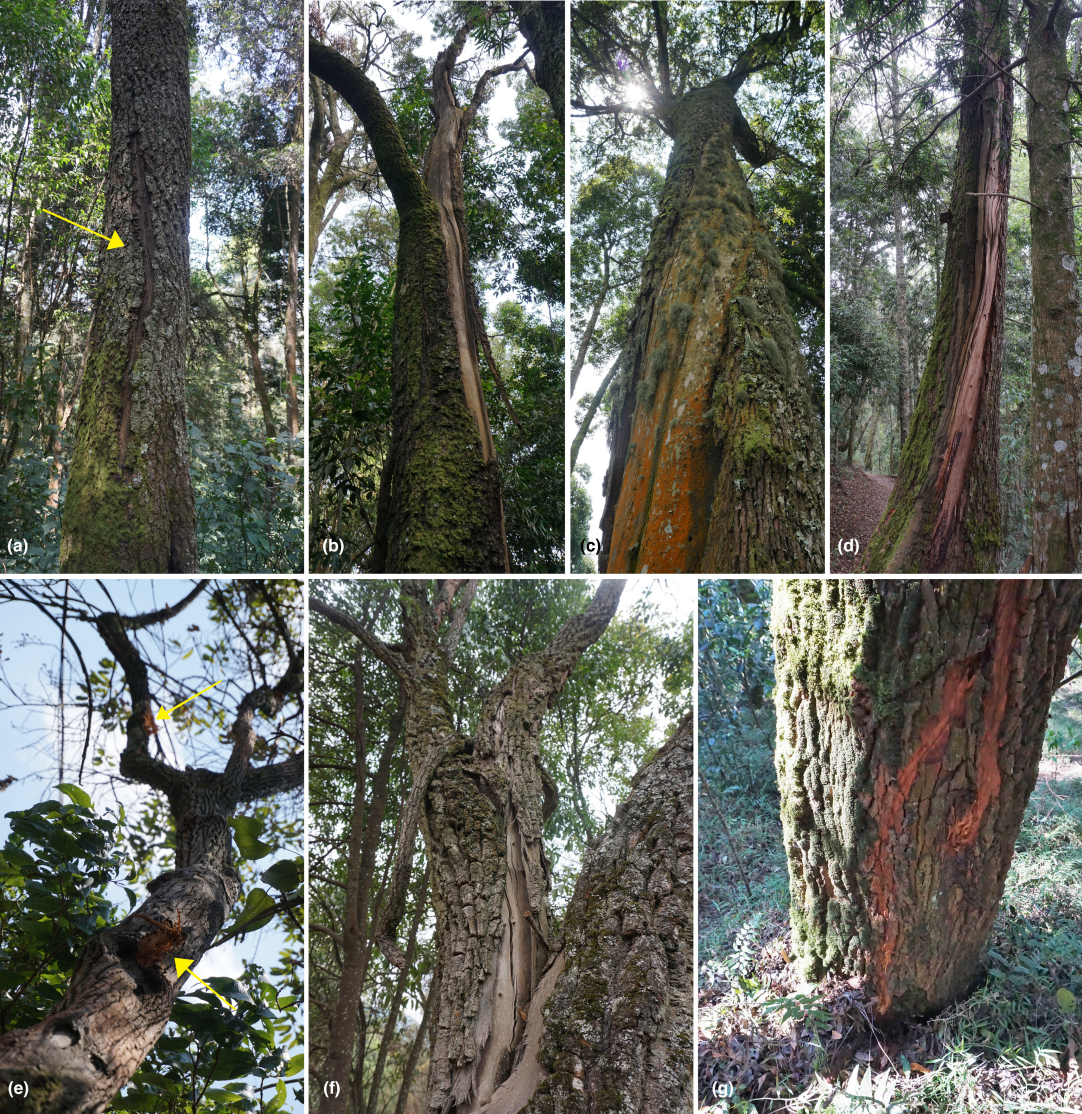
Seven trees with proposed lightning scars. These are characterized by a long furrow along the trunk and, around it, the removal of a portion of bark, which width can vary from few centimeters (a, e–g) to a few tens of centimeters (b–d). (a) *Faurea saligna* struck by lightning on April 7, 2015. The lightning killed a Gorilla and left a scar that at the time marked the trunk of the tree along its entire length, but which is now healing and is no longer than 2 m. (b) Dead *F. saligna* showing a long and straight lightning scar. (c) A wide and relatively old lightning scar spirals around the trunk of a *Ficalhoa laurifolia* tree, which is still alive. (d) The lightning scar on this *F. laurifolia* consists of a system of parallel fissures that develops longitudinally until the ground. (e) *Agauria salicifolia* struck by lightning on August 10, 2022, a few days before our survey. The marks left by lightning on this tree are peculiar since they are discontinuous. (f) Lightning scar on an *A. salicifolia*. According to witnesses, the tree was struck in 2015. (g) The only case of branched scar observed in our study. The tree, *A. salicifolia*, was struck on August 14, 2022, a few days before our survey. All photos by Bianca Zoletto.

Lightning in the tropics does not only kill gorillas but also impacts trees and forests. Observations suggest that lightning can shape forest dynamics (Anderson, 1964; Brünig, 1964) and tree species composition (Richards et al., 2021). A study on tree mortality on Krakatau, Indonesia, found that lightning strikes are a major cause of tree death, often resulting in single dead trees with split or charred trunks, and sometimes in several dead trees (Whittaker et al., 1998). A more recent study of strikes in Barro Colorado Island (BCI), Panama, estimated that each damaged on average 23.6 trees >10 cm in diameter, of which 5.5 died within a year (Gora et al., 2020). Moreover, lightning is an important cause of large tree mortality on BCI, with strikes estimated to cause the death of around half of all trees >60 cm in diameter (Yanoviak et al., 2020). Further studies in the same forest suggested that lightning strikes generate around 20% of annual gap area (area encompassing the crowns of dead and severely damaged canopy trees) and 13% of total woody biomass mortality (the sum of dead tree biomass and the cumulative necromass associated with crown dieback per strike) (Gora et al., 2021).

Although anecdotal observations of trees struck by lightning are common in the tropics (Anderson, 1964; Furtado, 1935; Magnusson et al., 1996), quantified evaluations remain difficult due to the challenge of consistently and objectively identifying impacted trees. Methods that have been used to detect lightning‐struck trees include camera‐based systems to locate lightning strikes in near real time (Yanoviak et al., 2017) and sensors that monitor electrical fields (Bitzer et al., 2013). However, these methods are expensive, challenging to implement, and cannot identify past strikes. Field‐based survey methods have been used to identify past strikes, and are relatively cheap and easy. However, for such field methods, it is important to define practical criteria that can be used to identify lightning strikes with reasonable confidence.

Previous studies have identified putative lightning strikes from the presence of perceived flashover damage, defined as the defoliation of the nearest branches in two (or more) adjacent trees and caused by electricity crossing an air gap between neighboring tree crowns (Yanoviak et al., 2017).

These methods do not make use of lightning scars because such scars appeared to be too infrequent. While commonly noted in temperate forests, scars are considered rare in the tropics as they have seldom been reported from surveys (Gora & Yanoviak, 2020; Parlato et al., 2020; Yanoviak et al., 2017). A pantropical study of tree mortality and damage, which involved over 150,000 trees, concluded that lightning damage was rare (Arellano et al., 2020)—with only a single tree in Pasoh (Malaysia) believed to bear a lightning scar (G. Arellano, personal communication). Our observations in the montane tropical forest of Bwindi Impenetrable National Park indicate otherwise. Bwindi Forest, in southwestern Uganda, ranges from 1190 to 2560 m a.s.l. and covers an area of 331 km^2^ on a steep and rugged terrain. The vegetation is classified as “medium altitude moist evergreen forest” and “high altitude sub‐montane forest” (Langdale‐Brown et al., 1964). The climate in Bwindi is equatorial with an annual mean rainfall of 1525 mm and an annual mean temperature of 18.4°. There are two rainfall peaks, from March to May and September to November (Institute of Tropical Forest Conservation [ITFC]; http://itfc.org/bwindi_introduction.htm). This is one of the areas with the highest lightning strike frequency in the world, with an estimated 10–20 lightning strikes per square kilometer per year (Christian et al., 2003), making it an interesting location for studying the effects of lightning.

During the summer of 2022, we walked 57 km of transect (corresponding to more than 90 ha of forest) and identified 44 lightning‐struck trees (over 20‐cm DBH) using a protocol based on the flashover criteria. Furthermore, we added to our dataset 16 trees that the Park Rangers or local people reported to us as having been struck by lightning (Appendix). These methods are not mutually exclusive: nine trees indicated by witnesses also showed flashover damage. Of these 60 struck trees, 18 trees showed what we judge to be clear evidence of lightning scars. We added two additional trees to our lightning‐struck trees dataset for which identification was based solely on the presence of lightning scars on their trunks. Since the data we collected is based on the detection of the selected indicators that can be attributed to lightning strikes, it is not possible to estimate the frequency of lightning strikes to trees in this forest and the number of strikes resulting in scars. Nevertheless, our observations indicate that in this equatorial mountain forest, lightning scars on lightning‐struck trees are common.

Although it is generally assumed that other phenomena may produce scars similar to those caused by lightning (e.g., improper closure of wounds, and the splitting of weak branch unions) our observations suggest that misinterpretation is likely to be uncommon. Of the 20 trees with lightning scars, 13 had two or more neighboring trees showing flashover damage, and for 11 trees eyewitnesses could confirm that they were struck by lightning (Appendix), including the tree associated with the gorilla death noted in our first paragraph. These cases provided multiple independent sources of evidence of strikes, allowing us to identify common characteristics of lightning scars: the scars appear as an open band, cleft, or wound, in most cases running for many meters from a large branch down the trunk to the ground (Figure 1b–d). Typically, these features are extended for nearly the full length of the stem. We assume that along the path taken by the powerful electric current, the high resistance of wood generates heat, killing tissues and producing steam that can force the bark to detach and the wood to be damaged, leading to the open wound (Figure 1f). The margins of the crack usually have an irregular and “scratched” appearance (Figure 1g). In addition, secondary cracks and fissures often form on the sides of the main scar (Figure 1d).

Because of these distinctive features, identifying lightning scars appears relatively easy and unambiguous. Nevertheless, scars do vary, ranging from hardly visible (Figure 1a,e) to extremely conspicuous (Figure 1b–d). In addition, while most scars consist of a continuous crack running to the ground (Figure 1b–d), in two cases we found discontinuous damage along the trunk (Figure 1e), and in one case we observed a branching of the scar (Figure 1g). Furthermore, it was found that mostly the scars develop in a straight line (Figure 1a,b,d), while sometimes they spiral around the trunk (Figure 1c). The extent of damage is also variable: often the tree survives, while in other cases it dies (7 out of our 20 trees). In one case, lightning broke the tree several meters above the ground, displacing the upper and scarred portion of the trunk a few meters from the base. The factors determining the differences in damage and scar formation, however, remain unknown. We speculate that location, exposure, and species characteristics are all involved. For example, a tree where the current can more readily reach the Earth via the stem's inner bark might be more likely to be scarred than in trees where alternative pathways are present (e.g., via lianas or neighboring stems). We also noticed that the scars tend to feature on trees with relatively hard and thick fissured bark than the thinner and smoother bark.

Based on our survey, we observe that lightning scars are common in this forest, and we present a set of criteria to define whether a scar can be ascribed to lightning with reasonable confidence: (i) from a distance, a lightning scar looks like a vertical strip of variable width and a minimum length of 2 m in which the bark has been removed. (ii) Along this strip the underlying tissues are exposed: either the cambium, which has an irregular and fibrous appearance (Figure 1g), or the sapwood, generally smoother and more regular (Figure 1d), are visible. (iii) The central part of the strip is typically characterized by either a vertical crack (or a system of parallel cracks) that often has a sinuous pattern and chipped edges or a shallow vertical groove. These criteria can be ambiguous to apply to the oldest scars where the bark has recovered and/or the exposed wood is already showing signs of additional rot or weathering.

As lightning scars appear relatively common and visible, we propose such scars may be an important feature to include in methods to detect and identify lightning strikes. We offer three arguments. First, scars are often the only visible damage caused by lightning to otherwise healthy trees. In our study, almost half of the trees with scars that we can confidently ascribe to lightning (based on witnesses) showed no flashover damage and would thus be omitted in conventional assessments. Taking these signs into consideration makes it possible to identify more strikes and may reduce bias due to only considering one form of damage involving at least three trees (i.e., one main tree and two or more indicating flashover damage). A more comprehensive identification of such strikes is useful to obtain a more general understanding of the phenomenon and its variability.

Second, flashover damage has limited persistence due to the growth of new foliage, which quickly replaces and obscures lightning damage. By contrast, lightning scars persist for years (at least 8 years as indicated by the oldest witnessed event included in our data). This persistence permits the identification of older events that otherwise would go unnoticed.

Third, the current best‐practice approach using flashover damage can sometimes be hard to apply with certainty. Many factors can damage trees in a way that may be similar to flashover damage (herbivory, attacks by pathogens, wind, drought, etc.). Difficulties arise also if deciduous species are present, and one cannot distinguish seasonal effects from lightning‐caused damage. Moreover, when trees have been dead for several weeks the pattern of defoliated branches between neighboring trees is obscured, which makes it more difficult to identify the flashover damage. For example, a cluster of 10 live trees in which we observe neighboring branches with clear and localized damage around a focal tree is a good indicator of lightning strike, but a cluster of 10 long‐dead trees is ambiguous.

We find that lightning scars are relatively common in at least one African forest. The wider significance of these findings is less clear, but our observations contrast with those from other tropical forests, where lightning damage appears scarce (Arellano et al., 2020) or where lightning damage is seen, but scars are absent (Yanoviak et al., 2017). These differences suggest that the nascent study of lightning requires the critical development of robust protocols that can account for substantial variation in the frequency and nature of any impacts, making comparisons across sites and among methods essential.

We conclude that, in this forest at least, lightning scars provide useful evidence of lightning strikes. This can help the study of these phenomena through broader datasets of trees struck by lightning with various degrees of damage. Furthermore, scars may be useful not only in detecting trees struck by lightning, but also in better understanding, this phenomenon and studying its effects. For example, dendrochronological and magnetic analyses of lightning scars could help date strikes, highlight any reduction in subsequent growth following a strike, or clarify how lightning impacts trees (Camarero et al., 2020). Such investigations would advance our understanding of lightning and its impacts.

## AUTHOR CONTRIBUTIONS

**Bianca Zoletto:** Conceptualization (equal); data curation (lead); writing – original draft (lead). **Masha T. van der Sande:** Writing – review and editing (equal). **Peter van der Sleen:** Writing – review and editing (equal). **Douglas Sheil:** Conceptualization (equal); writing – review and editing (equal).

## CONFLICT OF INTEREST STATEMENT

None declared.

## Data Availability

All the data used to write this paper are included in the paper itself, in the Appendix section.

## Appendix

Below is a list of the 62 sites in Bwindi Impenetrable National Park where we observed lightning‐damaged trees. Forty‐four of the sites were included because they fulfilled the flashover criteria, 16 sites are included because park rangers and local people reported to us they were struck by lightning, and two sites are included only because of the presence of lightning scars on their trunk.

For each site included in the dataset, the following information is provided: site code, the method we used to identify the site (detection method), the number of trees showing any type of damage that we ascribed to lightning, including flashover damage in the canopy or lightning scar on the trunk (lightning‐damaged trees), the number of dead trees present in the site (dead trees), the presence or absence of a lightning scar on one of the trees in the site (lightning scar), and the species of the tree that was primarily struck by lightning. Tree species marked with “*” are planted species.

**Table:**

Site code	Detection method	Lightning‐damaged trees	Dead trees	Lightning scar	Primarily struck tree species
T1	Witnessed	6	0	No	*Faurea saligna*
T2	Witnessed	3	1	No	*Agauria salicifolia*
T3	Flashover criteria	5	0	Yes	*F. saligna*
T4	Witnessed	1	0	No	*Psychotria mahone*
T5	Witnessed	7	2	No	*Markamia lutea*
T6 (Figure 1f)	Witnessed	1	1	Yes	*A. salicifolia*
T7	Flashover criteria	10	4	No	*Ficalhoa Laurifolia*
T8	Flashover criteria	10	0	No	*F. saligna*
T10	Flashover criteria	20	16	No	*F. laurifolia*
T11 (Figure 1d)	Witnessed	15	4	Yes	*F. laurifolia*
T12	Flashover criteria	3	1	No	*Olea capensis*
T13	Flashover criteria	6	2	No	*Mystroxylon aethiopicum*
T15 (Figure 1a)	Witnessed	1	0	Yes	*F. saligna*
T16	Flashover criteria	8	1	No	*F. saligna*
T18	Flashover criteria	6	0	No	*Syzygium guineense*
T23	Flashover criteria	6	1	No	*Nuxia congesta*
T26	Flashover criteria	7	3	No	*Bridelia micrantha*
T28	Flashover criteria	5	2	No	*B. micrantha*
T29	Flashover criteria	6	3	No	*Ilex mitis*
T30	Flashover criteria	4	0	No	*Chrysophyllum gorungosanum*
T31	Flashover criteria	4	0	No	*C. gorungosanum*
T32	Flashover criteria	3	1	No	*C. gorungosanum*
T35	Flashover criteria	11	3	Yes	*F. laurifolia*
T36	Flashover criteria	22	0	Yes	*S. guineense*
T37	Flashover criteria	5	1	No	*Harunyana madascariensis*
T38	Flashover criteria	8	0	No	*F. saligna*
T40	Flashover criteria	13	2	No	*Dichaetanthera corymbosa*
T43	Flashover criteria	4	4	No	*F. laurifolia*
T44	Flashover criteria	8	0	No	*Entandrophragma excelsum*
T46	Witnessed	2	0	No	*Prunus africana*
T48	Flashover criteria	12	2	No	*F. saligna*
T49	Flashover criteria	13	8	No	*F. laurifolia*
T51	Flashover criteria	16	13	Yes	*A. salicifolia*
T53 (Figure 1b)	Flashover criteria	11	3	Yes	*F. saligna*
T54	Flashover criteria	9	3	No	*F. laurifolia*
T55	Flashover criteria	7	3	Yes	*F. saligna*
T56	Flashover criteria	5	1	No	*F. saligna*
T59	Flashover criteria	7	3	No	*Polyscias fulva*
T60	Flashover criteria	4	0	No	*F. saligna*
T65	Flashover criteria	6	1	No	*F. saligna*
T67	Flashover criteria	10	2	No	*F. laurifolia*
T68	Flashover criteria	5	0	No	*A. salicifolia*
T69	Flashover criteria	3	0	No	*E. excelsum*
T70	Flashover criteria	9	1	No	*N. congesta*
T71	Flashover criteria	6	3	No	*H. madascariensis*
T72	Witnessed	5	1	Yes	*H. madascariensis*
T76	Flashover criteria	4	2	No	*H. madascariensis*
T77	Flashover criteria	6	1	No	*Sapiem ellitticum*
T78	Flashover criteria	5	1	Yes	*Newtonia buchananii*
T81	Witnessed	5	2	Yes	*F. laurifolia*
T82	Witnessed	1	0	Yes	*F. laurifolia*
T83	Flashover criteria	6	2	No	*F. laurifolia*
T85	Flashover criteria	6	3	No	*Ocotea usambarensis*
T92	Witnessed	2	1	Yes	*Pinus patula**
T93	Witnessed	7	1	Yes	*Cupressus lusitanica**
T96 (Figure 1c)	Presence of lightning scar	1	0	Yes	*F. laurifolia*
T97	Presence of lightning scar	1	0	Yes	*S. guineense*
T100	Witnessed	13	12	Yes	*P. patula**
T101 (Figure 1g)	Witnessed	6	0	Yes	*A. salicifolia*
T102 (Figure 1e)	Witnessed	1	1	Yes	*A. salicifolia*
T66	Flashover criteria	3	0	No	*Myrica salicifolia*
T39	Flashover criteria	3	0	No	*A. salicifolia*
